# Laparoscopic appendectomy in the elderly: our experience

**DOI:** 10.1186/1471-2482-13-S2-S22

**Published:** 2013-10-08

**Authors:** Alessia G Ferrarese, Valter Martino, Stefano Enrico, Alessandro Falcone, Silvia Catalano, Giada Pozzi, Silvia Marola, Mario Solej

**Affiliations:** 1 University of Turin, Department of Oncology, School of Medicine, Teaching Hospital "San Luigi Gonzaga", Section of General Surgery, Orbassano, Turin, Italy

## Abstract

**Background:**

Laparoscopic appendectomy for acute appendicitis is one of the most common surgical procedures performed in the world. We aimed to compare laparoscopic and open appendectomy in the elderly in our experience.

**Methods:**

We performed a retrospective review of elderly patients who underwent appendectomy for acute appendicitis from 1st of January 2006 to the 31st of July 2012. We analyzed 39 appendectomies in elderly patients: 20 procedures were performed using open technique (Group O) and 19 using laparoscopic technique (Group L).

**Results:**

In the analysis of intraoperative variables there was no statistically significant difference. In this study there was no statistically significant difference also in peri-operative variables.

**Conclusion:**

Laparoscopic appendectomy is a safe and feasible technique in acute appendicitis also in the elderly.

## Background

Laparoscopic appendectomy for acute appendicitis is one of the most common surgical procedures performed in the world [1-3]. The first surgeon performing a laparoscopic appendectomy was Semm in UK in 1983 [4]. Acute appendicitis in the elderly is a surgical disease that could create important diagnosis problems [5-9] as far as concerns the atypical presentation [3,10-18].

We aimed to present our experience about a series of laparoscopic appendectomies in elderly patients and analyze the feasibility of laparoscopic technique in comparison with open techinique.

### Methods

From the 1st of January 2006 to the 31st of July 2012 we performed 208 appendectomies in our division of General Surgery: 39 of these were performed in elderly patients (age > 65 yrs, 30 M 9 F). In the elderly group, 20 procedures were performed using open technique (Group O) and 19 using laparoscopic technique (Group L).

The analyzed variables were: sex, symptoms, CT or US evaluation, total hospital stay, hospital stay after and before the procedure, kind and duration of procedure, conversion to open procedure, drain and final pathological results.

Statistical proportions related to the dichotomic variables (gender distribution in the different patient groups, number of post-operative complications, conversion rate, number of drains, presence of fever, wall thickening, amount of effusion, presence of appendix perforation) were compared using Chi-square test and Fisher's exact test.

Continuous variables like age distribution, post-operative hospital stay time, surgery duration and several haematochemical characteristics (WBC, CRP) were expressed as average (range) and analyzed using the Mann-Witney U test. Patients distribution according to different surgical teams was confirmed. All statistical analyses were performed using R software (*version 2.6.2*), and a *p *value of less than 0.05 was considered statistically significant.

### Results

Table 1 shows demographic data of both groups.

**Table 1:** Demographic data

	Group O	Group L
Mean age (yrs)	76	74

Sex M%	16	14

F%	4	5

In the O group we performed a McBurney incision in 18 patients and a pararectal incision in 12 cases; all appendectomies were performed by loops. In laparoscopic appendectomy group in 11 cases we used the mechanical stapler (Table 2).

**Table 2:** Surgical data (ns: not significative)

	Group O	Group L
Mc Burney	18	

Pararectal incision	12	

Loops	20	8

Stapler		11

In intraoperative variables analysis there was no statistically significant difference (Table 3). In this study there was no statistically significant difference also in peri-operative variables (Table 4).

**Table 3:** Intraoperative data

	Group O	Group L	*p-value*
Mean operative time (min)	57	63	ns

Presence of perforated appendix	2	3	ns

Abdominal effusion	7	9	ns

**Table 4:** Peri-operative data data

	Group O	Group L	*p-value*
Pre-operative stay (days)	3,2	3	ns

Post-operative stay (days)	6,2	5	ns

Wound sepsis	0	0	ns

Total hospital stay (days)	10	8,4	ns

Drain apposition	14	11	ns

Post-operative complication	1	0	ns

Residents performed 3 surgical procedures (8,57%), and in 17 cases the resident was in equipe as second operator, with a total resident's presence in the Surgical Team of 51,28% of cases.

The follow-up was 19 months; the only post-operative complication was a wound infection in a open appendectomy, resolved with antibiotic therapy. There was no mortality.

## Conclusions

In our experience we assist to an inversion of surgical approach in acute appendicitis, with a gradual increase of laparoscopic procedures. In spite of slightly longer time of procedure, there was no significant difference in number of post-operative complications, number of drains, duration of surgical procedure and total hospital stay in laparoscopic appendectomy and open procedure [19-21]. Laparoscopic appendectomy is to be considered an advanced surgical procedure: anatomical variability and unpredictable difficulties make the procedure not standardizable.

We consider surgery approach more difficult in the elderly in some cases [22] but we also considered laparoscopic approach is, in general, a safe and feasible technique in acute pathology [23] and a safe approach also in the elderly [24,25]. Laparoscopic appendectomy for acute appendicitis is a gold standard technique also in the elderly.

## Competing interests

The authors declare that they have no competing interests.

## Authors' contributions

AGF: conception and design, interpretration of data, given final approval of the version to be published.

VM: critical revision, interpretation of data, given final approval of the version to be published.

MS: acquisition of data, drafting the manuscript, given final approval of the version to be published.

AF: acquisition of data, drafting the manuscript, given final approval of the version to be published.

SC: acquisition of data, drafting the manuscript, given the final approval of the version to be published.

GP: acquisition of data, drafting the manuscript, given the final approval of the version to be published.

SM: critical revision, interpretation of data, given final approval of the version to be published

SE: conception and design, interpretration of data, given final approval of the version to be published

## Declarations

Funding for this article came from personal funds.

This article has been published as part of *BMC Surgery *Volume 13 Supplement 2, 2013: Proceedings from the 26th National Congress of the Italian Society of Geriatric Surgery. The full contents of the supplement are available online at http://www.biomedcentral.com/bmcsurg/supplements/13/S2
